# *Nef* as a Proliferative Factor for Kidney Epithelial Cells in HIV-Associated Nephropathy

**DOI:** 10.4137/cmo.s661

**Published:** 2008-10-30

**Authors:** Krishna K. Ratnam, Cijiang John He, Paul Klotman

**Affiliations:** Department of Medicine, Mount Sinai School of Medicine, New York, New York, U.S.A

**Keywords:** HIV-associated nephropathy, *nef*, podocyte injury, src kinase, signal tranducer and activator of transcription 3, mitogen-activated protein kinase

## Introduction

HIV-associated nephropathy (HIVAN) is the leading cause of end-stage renal disease in patients with HIV-1 infection. While HIVAN involves all portions of the kidney, marked changes in podocyte proliferation and dedifferentiation are central to the pathogenesis of this disease. This review focuses on identifying the genes and signaling pathways that trigger terminally differentiated quiescent podocytes to lose their normal markers of maturity and reenter the cell cycle. The parallels between these alterations to malignant transformation are quite striking.

## HIVAN and Features of Podocyte Injury

HIV-associated nephropathy (HIVAN) is the leading cause of chronic kidney failure in patients infected with HIV (USRDS 2005). The majority of patients with HIVAN are of African descent and HIVAN is the third leading cause of end-stage renal disease in African-Americans between the ages of 20–64 years old (Winston et al. 1998; Schwartz et al. 2005). Typically, HIVAN develops in the setting of advanced HIV-1 infection. HIVAN clinically has been characterized by proteinuria, often in the nephrotic range, and progressive renal insufficiency (Rao et al. 1984; Carbone et al. 1989; Burns et al. 1997; Winston et al. 1999; Ross and Klotman, 2002). Renal biopsy is required to make the definitive diagnosis of HIVAN. Pathologic findings include microcystic dilatation of renal tubules, collapsing focal segmental glomerulosclerosis, and leukocyte infiltration and fibrosis of the renal interstitium (D’Agati et al. 1989; D’Agati and Appel, 1998; Ross et al. 2001). Prominent proliferation of renal tubular and visceral epithelial cells (podocytes) is a characteristic feature of this disease. Podocytes exhibit hypertrophy as well as hyperplasia in HIVAN (D’Agati and Appel, 1997; Lu et al. 2007).

The HIV virus directly infects tubular cells and podocytes leading to the development of HIVAN. Not surprisingly, the HIV-1 transgenic mouse (Tg26 mice) model in which renal cells express viral proteins has greatly advanced our knowledge of the pathogenesis of HIVAN. The Tg26 mice develop proteinuria, progressive renal failure, and renal pathologic findings identical to HIVAN patients (Dickie et al. 1991; Kopp et al. 1992). Reciprocal renal transplant between wild-type and Tg26 mice definitively demonstrated the direct link between HIV expression at the kidney to the renal phenotype. HIVAN develops in transgenic kidneys transplanted into nontransgenic littermates, but not in normal kidneys transplanted into transgenic littermates (Bruggeman et al. 1997). In studies in macaques, investigators isolated simian-human immunodeficiency virus (SHIV) from glomeruli (Stephens et al. 2000). HIV-1 RNA has also been detected by RNA in situ hybridization from renal biopsy specimen in HIV-seropositive patients (Bruggeman et al. 2000). HIV-1 has been detected in several portions of the nephron, including glomerular visceral and parietal epithelial cells and tubular cells (proximal tubule, thick ascending limb of loop of Henle, and collecting duct) (Bruggeman et al. 2000; Ross et al. 2001).

Expression of HIV-1 in podocytes is critical to the development of disease pathogenesis. Podocytes line the outer aspect of the glomerular basement membrane and serve as the final barrier to protein loss. A clear link exists between injury to these cells and the development of the HIVAN phenotype. Using the Nphs1 promoter in the transgenic mouse model, researchers demonstrated that selective podocyte expression of HIV-1 proviral DNA with mutations in *gag, pol*, and *env* leads to the development of massive proteinuria, severe collapsing FSGS, and microcystic tubular dilatation (Zhong et al. 2005). This study conclusively illustrated that podocyte-restricted HIV-1 gene expression was sufficient for development of collapsing glomerulosclerosis.

Unlike other glomerular diseases, HIV-1 infection triggers both podocyte proliferation and dedifferentiation (Lu et al. 2007). Podocytes are terminally differentiated post-mitotic cells that, under normal conditions, have lost their ability to proliferate (Sunamoto et al. 2003). Normal mature podocytes remain in a quiescent state and express cyclin-dependent kinase (CDK) inhibitors p27 and p57 and do not express markers of proliferation (cyclin A, cyclin D, and Ki-67) (Barisoni et al. 2000a; Barisoni et al. 2000b; Shankland and Wolf ). The collapse of glomerular capillaries in HIVAN, however, occurs in the setting of marked podocyte proliferation (Barisoni et al. 1999; Barisoni et al. 2000a; Barisoni et al. 2000b). This podocyte hyperplasia is driven by cell cycle dysregulation. In HIVAN, podocytes exhibit increased expression of Ki-67, cyclin A, and cyclin D and decreased expression of CDK inhibitors p27 and p57 (Barisoni et al. 2000a; Shankland et al. 2000). The net effect of these changes is the reentry of the podocyte into the cell cycle, resultant G1 to S phase progression and, thus, cell proliferation. In subsequent paragraphs, I will present evidence that the effects on cell cycle dysregulation have been mapped to the expression of the HIV gene *nef*. Indeed, the growth rate of podocytes as measured by ^3^H thymidine uptake was threefold greater in the HIV-1 Tg podocytes compared to nontransgenic podocytes (Schwartz et al. 2001).

HIVAN is also accompanied by podocyte dedifferentiation. This is characterized by the loss of several podocyte differentiation markers, such as Wilm’s tumor (WT-1), synaptopodin, podocalyxin, common acute lymphoblastic leukemia antigen (CALLA), and ezrin (Barisoni et al. 1999; Barisoni et al. 2000a; Barisoni et al. 2000b). The loss of synaptopodin staining is the most specific for HIVAN on immunohistochemical analysis of kidney biopsy specimen (Barisoni et al. 1999). Dedifferentiation of podocytes appears to be unique to HIVAN as these markers are not lost in other glomerular diseases associated with the nephrotic syndrome (e.g. minimal change disease and membranous nephropathy) (Barisoni et al. 1999). Podocytes from the HIV Tg26 mouse model exhibit a loss of contact inhibition. Additionally, podocytes from the HIVAN model demonstrate anchorage independent growth of colonies in soft agar *in vitro* (Schwartz et al. 2001; Shah et al. 2006). This is in contrast to the normal inhibition of growth of immortalized visceral epithelial cells from nontransgenic mice upon reaching confluence.

## Role of *nef* in the Pathogenesis of HIVAN

The HIV-1 genome consists of nine genes: three structural genes (*gag, pol, env*), two regulatory genes (*tat* and *rev*), and four accessory genes (*vif, vpr, vpu*, and *nef* ). In HIV-1 infected hosts, the 27-kDa protein Nef is at an early phase in the viral replication cycle and performs several functions (Li et al. 2005). These include enhancement of viral replication and infectivity, down-regulation of cell surface proteins such as CD4 and major histocompatibility complex (MHC) class I and II, alteration of signal transduction pathways, and modulation of endosome trafficking pathways (Fackler and Baur, 2002; Wei et al. 2003; Li et al. 2005; Zuo et al. 2006). *In vitro* and *in vivo* data support a critical role for Nef in the development of podocyte proliferation and dedifferentiation seen in HIVAN.

Husain et al. infected conditionally immortalized podocytes with HIV-1 proviral constructs with premature stop codons inserted into the individual *env, vif, vpr, vpu, nef,* or *rev* genes (Husain et al. 2002). Constructs expressing all of the above genes led to marked podocyte proliferation and anchorage independent growth (growth of colonies in soft agar). Additionally, no change in growth parameters was observed in cells infected with Δ*env*, Δ*vif*, Δ*vpr*, Δ*vpu*, or Δ*rev* viruses. Proliferation and colony formation in soft agar, however, were abrogated in podocytes infected with a Δ*nef* virus. Finally, infection with a construct expressing the nef gene alone was sufficient to induce podocyte proliferation, loss of contact inhibition, and anchorage independent growth *in vitro* (Husain et al. 2002).

Sunamoto et al. then utilized the same HIV-1 mutant proviral constructs lacking either *env, vif, vpr, vpu, nef,* or *rev* genes to establish that *nef* induces the dedifferentiation of podocytes in HIVAN (Sunamoto et al. 2003). In fact, constructs containing *nef* alone are sufficient to suppress the markers of podocyte maturity synaptopodin, CALLA, and ezrin. Additionally, the presence of *nef* alone also led to decreased expression of the CDK inhibitors (p21 and p27) which normally prevent mature podocytes from reentering the cell cycle (Sunamoto et al. 2003). The presence of *nef* was also sufficient to increase the expression of the proliferation markers Ki-67, cyclin A, and cyclin E (Sunamoto et al. 2003). Only podocytes infected with constructs lacking *nef* demonstrated a mature pattern of differentiation markers and lack of proliferation. These *in vitro* data show that Nef is the major gene product of HIV-1which drives the changes in podocyte dedifferentiation and induces reentry into the cell cycle and subsequent visceral epithelial cell hyperplasia.

In vivo data has confirmed *nef* ‘s critical role in the development of the HIVAN phenotype. Hanna et al., by mutating HIV genes in transgenic mice, demonstrated that *nef* expression under a human CD4 promoter induced interstitial nephritis and tubular atrophy and dilatation as seen in HIVAN (Hanna et al. 1998). Given the use of the CD4 promoter, however, *nef* expression in podocytes was likely low and these mice failed to develop glomerular disease. A study by Kajiyama et al. illustrated the onset of glomerulosclerosis and tubular dilatation by 6 weeks of age in transgenic mice infected with the HIV-1 provirus with an inactivated *nef* coding sequence (Kajiyama et al. 2000). When this transgenic line was crossed with transgenic mice expressing *nef* alone, offspring developed more severe glomerulosclerosis. Husain et al. conducted a subsequent study with the transgenic mouse model to examine the role of podocyte-specific *nef* expression *in vivo* (Husain et al. 2005). *Nef* expressing podocytes had a loss of the differentiation marker synaptopodin and increased expression of the proliferation marker Ki-67 compared to control mice. *Nef* expressing mice, however, failed to develop significant proteinuria, had normal histology by light microscopy, and had only mild podocyte effacement on electron microscopy. These *in vivo* studies illustrate that *nef* plays a key role in the pathogenesis of glomerular and tubulointerstitial injury seen in HIVAN. Additionally, there are likely other as of yet unknown factors that also contribute to the complex phenotype seen in HIVAN. For example, HIV-1 genes *nef* and *vpr* may act synergistically in HIVAN pathogenesis. While mice with podocyte-specific expression of *nef* or *vpr* develop glomerulosclerosis, mice expressing both *nef* and *vpr* at podocytes develop more severe sclerosis (Zuo et al. 2006).

## *Nef* ‘s Effect on Downstream Signaling Pathways

**Figure 1:**
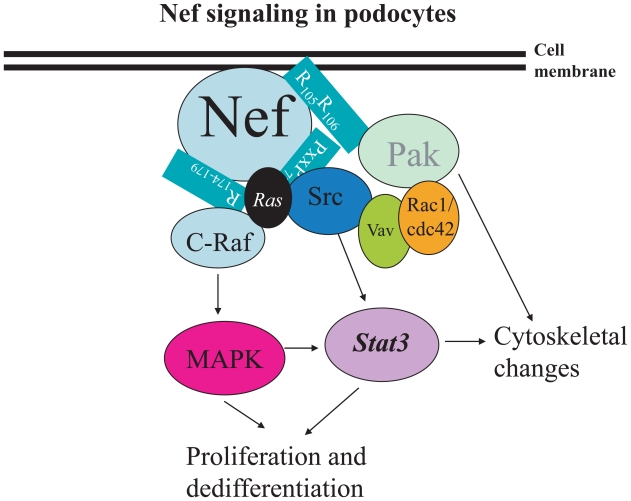
*Nef* binds to Src kinase, c-Raf, p21-activated kinase (PAK2), and the guanine nucleotide exchange factor Vav. The *nef* domains that serve as binding motifs for these interactions are outlined in this figure. Via a Src-dependent pathway, nef activates both the Stat3 and Ras-cRaf mitogen-activated protein kinase (MAPK) 1, 2 pathways. Activation of these pathways leads to podocyte proliferation and dedifferentiation. Nef, via its R105106 domain, activates PAK2 and mediates alterations in the actin cytoskeleton in podocytes.

Given *nef* ‘s intricate involvement in furthering proliferation and dedifferentiation of visceral epithelial cells at the glomerulus, several studies have started to unravel the downstream signaling pathways through which *nef* effects these changes. These pathways and identified biological effects *nef* has on the podocyte are summarized in Figure 1. The C-terminal loop of *nef* mediates viral replication and interacts with trafficking molecules to down-regulate expression of CD4 and MHC I (Schwartz et al. 1996; Piguet et al. 1999). Nef contains a polyproline helix type II which forms a (PxxP)_3_ sequence cluster (Lee et al. 1996; Geyer et al. 2001). This sequence mediates the interaction between the SH3 domain of Src family kinases and the guanine nucleotide exchange factor Vav (Lee et al. 1996; Geyer et al. 2001). Nef also interacts with several other cellular proteins, such as protein kinase C, mitogen-activated protein kinase (MAPK), Raf1, p21-activated kinase (PAK), pI3K, Vav1, and Vav2 (Arold and Bauer, 2001).

The Src family kinases are key potentiators of cell proliferation, cell to cell adhesion, and cell motility (Martin, 2001). These nonreceptor tyrosine kinases mediate these effects by leading to phosphorylation of proteins, which then in turn activate signaling pathways and other protein-protein interactions. Members of the Src family kinases include Src, Hck, Fgr, Lck, Lyn, and Yes. Studies have implicated several of these kinases in podocyte pathophysiology. For example, Fyn binds and phosphorylates nephrin and Δ Fyn leads to the coarsening of podocyte foot processes (Lahdenpera et al. 2003; Verma et al. 2003).

Src activation by Nef leads to activation of the transcription factor signal transducer and activator of transcription 3 (Stat3) (Akira, 2000). Phosphorylated Stat3 dimerizes, moves into the nucleus, and serves as a transcription factor for genes involved in cell growth, differentiation, and apoptosis. Stat3 activation has been demonstrated in both the developing kidney and in renal cell carcinoma (Barasch et al. 1999; Horiguchi et al. 2002). Stat3, after activation by Src in hematopoetic cells, causes upregulation of the cell proliferation marker cyclin E (Odajima et al. 2000).

Activation and signaling of the MAPK family plays a role in mitogenesis and cell differentiation. Nef can activate this downstream signaling pathway via Raf and Src activation (Tian et al. 2000). MAPK signaling is upregulated in several kidney diseases, such as rodent models of proliferative anti-glomerular glomerulonephritis, ischemia-reperfusion injury, renal cell carcinoma, and autosomal-dominant poly-cystic kidney disease (Oka et al. 1995; Bokemeyer et al. 1997; Nagao et al. 2003; Yang et al. 2003).

He et al. completed studies in immortalized and differentiated podocytes to establish a link between *nef* and downstream signaling pathways *in vitro* (He et al. 2004). Podocytes infected with *nef* have increased Src kinase activity and Stat3 phosphorylation compared to controls. Use of PP2, an inhibitor of Src kinase phosphorylation, blocks both Src kinase and Stat3 phosphorylation and, thus, confirms the configuration of the downstream signaling pathway. Expression of *nef* in podocytes also activates the Ras-c-Raf-MAPK1, 2 pathway (He et al. 2004). A dominant negative mutant form of Src kinase (Src-DN) abrogated *nef*-induced phosphorylation of Stat3 and MAPK1, 2. Src-DN inhibited podocyte expression of the proliferation marker cyclin E and restored markers of mature podocyte differentiation. Therefore, this dominant negative mutant data established Src kinase as an upstream activator of both Stat3 and MAPK1, 2 in mediating *nef*-induced podocyte phenotypic changes of proliferation and dedifferentiation (He et al. 2004). Use of small interference RNA to reduce *nef* expression decreased Stat3 and MAPK1, 2 phosphorylation, cyclin E expression, and podocyte cell proliferation (He et al. 2004). Finally, inhibition of MAPK1, 2 phosphorylation and a dominant negative mutant of Stat3 partially reversed the effects of *nef* on the podocyte proliferation and dedifferentiation (He et al. 2004). The absence of a complete phenotypic reversal with Stat3 and MAPK1, 2 inhibition indicate that yet undiscovered pathways downstream from Nef or Stat3 may also contribute to the changes at the podocyte in HIVAN.

Two Nef domains (PxxP and R_105_R_106_) are binding sites for the SH3 domain of the Src kinase family and PAK2. Using immortalized murine podocytes, mutations of Nef in the PxxP region have been shown to have the following effects *in vitro*: inhibition of Stat3 and MAPK1, 2 phosphorylation, reduction of cyclin E expression, restoration of synaptopodin expression, and reversal of Nef-induced cell proliferation (He et al. 2004). While mutation of the R_105_R_106_ domain did increase synaptopodin expression, mutation at this site failed to change phosphorylation of Stat3 or MAPK1, 2 and only minimally decreased cell proliferation (He et al. 2004). The data presented by He et al. illustrate that the PxxP motif of Nef is the site responsible for Nef-induced Stat3 and MAPK1, 2 phosphorylation and the downstream cellular signaling effects leading to podocyte proliferation and dedifferentiation. While the R_105_R_106_ motif may exert some of Nef’s effects leading to the HIVAN phenotype in podocytes, it is likely through a signaling cascade independent of Stat3 and MAPK1, 2 activation. A summary of known pathways of *nef* signaling and its downstream effectors is presented in Figure 1.

We confirmed these signaling pathways are critical to mediating Nef’s effect on the HIVAN phenotype *in vitro*. Immunostaining for phospho-Stat3 is markedly elevated in the glomeruli of transgenic mice with podocyte-specific *nef* expression compared to control littermates (Husain et al. 2005). Also, in human kidney biopsy specimen from patients with HIVAN, immunostaining demonstrated increased staining of phospho-Stat3 and phospho-MAPK1, 2 compared to controls. The control patients in this experiment included HIV-1 patients without HIVAN and non-HIV infected patients with minimal change disease, idiopathic collapsing FSGS, or classic FSGS (He et al. 2004). Additionally, in the Tg26 mouse, mutating the PxxP SH3-binding domain of *nef* generated mice that no longer exhibited the HIVAN phenotype (Omori et al. 2000). This study provided further *in vivo* evidence that *nef* induces the myriad of renal cortical abnormalities that characterize the HIVAN phenotype via activation of the Src family of tyro-sine kinases.

Vincent et al. have proposed that *nef* interaction and activation of PAK2 in the murine model contributes to the HIVAN phenotype (Renkema et al. 1999; Arora et al. 2000; Vincent et al. 2006). Activated PAK has been proposed to play a role in cell cytoskeletal reorganization, MAPK signaling cascades, pro and anti-apoptotic effects, and virion infectivity (Wiskerchen and Cheng-Meyer, 1996; Bagrodia et al. 1997; Rudel and Bokoch, 1997; Rudel et al. 1998; Sells et al. 1999; Schurmann et al. 2000; Jakobi et al. 2001). HIV transgenic mice that express *nef* alleles but lacking the Nef—PAK2 association do not exhibit kidney disease. This identifies Nef binding and activating PAK2 as critical to the development of HIVAN. Activation of PAK2 alone, however, is not sufficient to develop kidney disease. Transgenic mice expressing *nef* mutants that were able to bind and activate PAK2 failed to exhibit kidney disease (Vincent et al. 2006).

## Conclusion

HIVAN is characterized by marked proteinuria, renal insufficiency and characteristic pathologic changes on kidney biopsy. Marked podocyte proliferation and dedifferentiation occurs within the collapsing glomerulopathy found in HIVAN. These podocyte changes are not seen in other glomerular diseases. HIV-1 Nef is the critical protein mediating reentry of podocytes into the cell cycle and down-regulation of mature podocyte differentiation markers. *In vitro* and *in vivo* evidence indicates that Nef induces these changes in the podocyte via Src kinase-dependent activation of Stat3 and MAPK1,2 signaling pathways. Additionally, Nef interaction with PAK2 may contribute to some of the features of the HIVAN phenotype. There are still several downstream targets that have yet to be identified which potentiate the development of podocyte proliferation and dedifferentiation. Evidence also indicates that Nef may activate other pathways, in addition to Stat3 and MAPK1, 2, as part of the pathogenesis of HIVAN. Continued studies are needed to identify downstream effectors of Nef and therapeutic interventions that may abrogate the activity of these signaling pathways.
